# Molecular response and quality of life in chronic myeloid leukemia patients treated with intermittent TKIs: First interim analysis of OPTkIMA study

**DOI:** 10.1002/cam4.3778

**Published:** 2021-02-16

**Authors:** Michele Malagola, Alessandra Iurlo, Elisabetta Abruzzese, Massimiliano Bonifacio, Fabio Stagno, Gianni Binotto, Mariella D’Adda, Monia Lunghi, Monica Crugnola, Maria Luisa Ferrari, Francesca Lunghi, Fausto Castagnetti, Gianantonio Rosti, Roberto M. Lemoli, Rosaria Sancetta, Maria Rosaria Coppi, Maria Teresa Corsetti, Giovanna Rege Cambrin, Atelda Romano, Mario Tiribelli, Antonella Russo Rossi, Sabina Russo, Lara Aprile, Monica Bocchia, Lisa Gandolfi, Mirko Farina, Simona Bernardi, Nicola Polverelli, Aldo M. Roccaro, Antonio De Vivo, Michele Baccarani, Domenico Russo

**Affiliations:** ^1^ Unit of Blood Disease and Stem Cell Transplantation Department of Clinical and Experimental Sciences University of Brescia ASST‐Spedali Civili Brescia Italy; ^2^ Hematology Division Foundation IRCCS Ca Granda Ospedale Maggiore Policlinico Milan Italy; ^3^ Division of Hematology S. Eugenio Hospital ASL ROMA2 Tor Vergata University Roma Italy; ^4^ Department of Medicine Section of Hematology University of Verona Verona Italy; ^5^ Department of Hematology University of Catania Catania Italy; ^6^ Hematology and Clinical Immunology Department of Medicine Padua School of Medicine Padua Italy; ^7^ Division of Hematology ASST‐Spedali Civili di Brescia Brescia Italy; ^8^ Division of Hematology Department of Translation Medicine University of Eastern Piedmont Novara Italy; ^9^ Hematology Unit and BMT Center Azienda Ospedaliero Universitaria Parma Parma Italy; ^10^ Hematology and Bone Marrow Transplant Unit ASST Papa Giovanni XXIII Bergamo Italy; ^11^ Hematology and Bone Marrow Transplantation (BMT) Unit San Raffaele Scientific Institute Milan Italy; ^12^ Hematology Unit Department of Experimental, Diagnostic and Specialty Medicine (DIMES) S. Orsola‐Malpighi University Hospital University of Bologna Bologna Italy; ^13^ IRCCS Istituto Romagnolo per lo Studio dei Tumori (IRST) "Dino Amadori" Meldola Italy; ^14^ Clinic of Hematology University of Genoa Ospedale Policlinico S. Martino IRCCS Genoa Italy; ^15^ Hematology Unit Dell'Angelo Hospital Venezia‐Mestre Italy; ^16^ Haematology and BMT Unit "Antonio Perrino" Hospital Brindisi Italy; ^17^ Hematology Division Azienda Ospedaliera Santi Antonio e Biagio e Cesare Arrigo Alessandria Italy; ^18^ Medicina Interna a Indirizzo Ematologico Ospedale San Luigi Orbassano Italy; ^19^ IRCCS Regina Elena National Cancer Institute‐Rome Roma Italy; ^20^ Division of Hematology and BMT Department of Medical and Morphological Researches University of Udine Udine Italy; ^21^ Hematology and Transplants Unit University of Bari Bari Italy; ^22^ Division of Hematology Dipartimento di Patologia Umana dell'Adulto e dell'Età Evolutiva Policlinico G Martino University of Messina Messina Italy; ^23^ SC Ematologia, Ospedale S.G.Moscati Taranto Italy; ^24^ Hematology Unit Department of Medicine, Surgery and Neuroscience Azienda Ospedaliera Universitaria Senese University of Siena Siena Italy; ^25^ CREA Laboratory (Hematological‐Research AIL Centre) ASST‐Spedali Civili Brescia Brescia Italy; ^26^ Clinical Research Development and Phase I Unit ASST‐Spedali Civili Brescia Brescia Italy; ^27^ University of Bologna Bologna Italy

**Keywords:** chronic myeloid leukaemia, intermittent, quality of life, tyrosine kinase inhibitor

## Abstract

**Background:**

Intermittent treatment with TKIs is an option for the great majority (70%–80%) of CML patients who do not achieve a stable deep molecular response and are not eligible for treatment discontinuation. For these patients, the only alternative is to assume TKI continuously, lifelong.

**Methods:**

The Italian phase III multicentric randomized OPTkIMA study started in 2015, with the aim to evaluate if a progressive de‐escalation of TKIs (imatinib, nilotinib, and dasatinib) is able to maintain the molecular response (MR^3.0^) and to improve Health Related Quality of Life (HRQoL).

**Results:**

Up to December 2018, 166/185 (90%) elderly CML patients in stable MR^3.0^/MR^4.0^ completed the first year of any TKI intermittent schedule 1 month ON and 1 month OFF. The first year probability of maintaining the MR^3.0^ was 81% and 23.5% of the patients who lost the molecular response regained the MR^3.0^ after resuming TKI continuously. Patients’ HRQoL at baseline was better than that of matched peers from healthy population. Women was the only factor independently associated with worse baseline HRQoL (*p* > 0.0001). Overall, global HRQoL worsened at 6 (*p* < 0.001) but returned to the baseline value at 12 months and it was statistically significantly worse in women (*p* = 0.001).

**Conclusions:**

De‐escalation of any TKI by 1 month ON/OFF schedule maintains the MR^3.0^/MR^4.0^ in 81% of the patients during the first 12–24 months. No patients progressed to accelerated/blastic phase, all the patients (23.5%) losing MR^3.0^ regained the MR^3.0^ and none suffered from TKI withdrawn syndrome. The study firstly report on HRQoL in elderly CML patients moving from a continuous daily therapy to a de‐escalated intermittent treatment.

## Graphical abstract

The 1‐year probability of maintaining the MR3.0 while on intermittent 1 month ON/OFF TKI therapy was 81%. Patients’ QoL at baseline was better than that of matched peers from healthy population. Diarrhea significantly improved at 6 and 12 months (*p* = 0.022) and fatigue worsened at 6 (*p* = 0.001) and 12 months (*p* = 0.022). Overall, global QoL worsened at 6 (*p* < 0.001) but returned to the baseline value at the 12 months

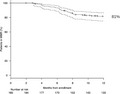

## 1 INTRODUCTION

Tyrosine Kinase Inhibitors (TKIs) significantly improved the life expectancy of patients with Philadelphia‐positive Chronic Myeloid Leukaemia (CML).^1^, ^2^, ^3^

The current treatment strategy with TKIs aims to prevent CML progression to accelerated/blastic phase (AP/BP) and to access drug discontinuation and treatment‐free remission (TFR). Molecular response (≤0.1% BCR‐ABL1%IS) is achieved in 80–90% of patients and 30–50% of them obtain deep molecular response (DMR) (≥MR^4.0^ ≤0.01% BCR‐ABL1%IS or 10.000 copies of transcript as minimum sum of reference gene). Patients in stable DMR have access to TFR, but, invariably, half of them loses molecular response with need of restarting treatment.^4^

Although no more than 20%–25% of the whole CML patients population can get and maintain the TFR,^5^ TKIs discontinuation has become the current paradigm of CML management and TFR the main goal of TKI therapy.^6^, ^7^, ^8^, ^9^

This current approach, nowadays recommended,^10^, ^11^ is far from being optimal and, in absence of concrete strategies to increase the number of deep molecular responders, it makes approximately 80% of the patients to have no alternative but to continue the daily treatment lifelong.^6^, ^12^, ^13^, ^14^

For these latter patients, this expectation raises a number of important questions concerning the adherence and tolerance to therapy, including the late and unexpected side effects, the quality of life, and the sustainable costs of therapy. These questions are particularly relevant in the CML elderly population, in whom these issues are likely to be not only dose dependent but also age related.^15^

Furthermore, these questions are also clinically and socially relevant because it is known that the incidence of CML progresses with age and, in the next years, the prevalence of CML in elderly population is expected to increase.^16^, ^17^, ^18^, ^19^

The previous phase II INTERIM study^20^ demonstrated that a policy of intermittent imatinib treatment (1 month ON–one month OFF) in elderly CML patients is feasible and successful, at long term. After 6 years of follow up, neither progression to blastic phase nor CML‐related deaths were recorded, the patients who had lost the complete cytogenetic response (CCyR) regained the CCyR after resuming imatinib continuously and 60% are on intermittent treatment in CCyR and MR^3.0^ or MR^4.0^.^21^ Furthermore, grade I‐II side effects disappeared in more than 50% of the patients on intermittent treatment.

The European Forum for Good Clinical Practice (EFGCP) pointed out the need to increase participation of elderly people in clinical trials, particularly in the case of patients with hematologic malignancies, where health‐related quality of life (HRQoL) issues have been understudied.^22^ Since little data exist on the effect of TKIs on quality of life in CML patients,^23^, ^24^ new specific clinical trials, which also include HRQoL, may provide the lacking information in elderly CML patients.

To this purpose, in July 2015, an Italian prospective multicentric randomized phase III trial was started with the aim to validate the policy of the intermittent de‐escalation treatment and to explore the impact of this strategy on the HRQoL. In this first interim report, we focused on the patients who, by intention to treat, have completed the first year of the study, to achieve information on maintenance of MR^3.0^/MR^4.0^ molecular response and also HRQoL.

## 2 PATIENTS AND METHODS

### 2.1 OPTkIMA study design

OPTkIMA study is an ongoing Italian phase III multicentric randomized study, where a “fixed” intermittent administration (1 month ON/OFF) of TKI (control arm), the same of the previous INTERIM Study, is compared with a “progressive” intermittent administration (1 month ON–1 month OFF for the 1^st^ year; 1 month ON–2 months OFF for the 2nd year; and 1 month ON–3 months OFF for the 3^rd^ year) (experimental arm). The study was approved by the local Ethical Committees of all the participating Centers, and was registered at ClinicalTrials.gov (NCT02326311).

Upon signing of written informed consent, chronic‐phase (CP) Ph+ CML patients older than 60 years and in MR^3.0^ or MR^4.0^ after ≥2 years of daily treatment with imatinib (IM), nilotinib (NIL), or dasatinib (DAS) have been randomized 1:1 to receive “fixed” or “progressive” intermittent administration. Randomization has been stratified by type of TKI (IM, NIL, or DAS) and by depth of molecular response (MR^3.0^ or MR^4.0^), according to the IS, as recommended by the International Experts Panel's Guide Lines.^21^ IM, NIL, or DAS have been administered intermittently at the same daily dose given at the time of the enrollment.

The study aims to validate “fixed” intermittent administration (1 month ON/OFF) of TKI, previously explored in the INTERIM Trial,^21^ and to evaluate if a “progressive” increase in intermittent treatment discontinuation until 3 months is able to maintain the MR^3.0^ / MR^4.0^ molecular response and improve HRQoL outcomes. Patients’ self‐reported European Organization for Research and Treatment of Cancer (EORTC) outcome measures have been assessed throughout the 3 years follow‐up period.

Molecular monitoring was performed according to the 2013 ELN guidelines every 3 months by RT‐qPCR on peripheral blood.^25^ In case of MR^3.0^ loss, checked in two monthly consecutive RT‐qPCR analysis, patients were planned to resume TKI daily and continued to be followed.

### 2.2 Definition of molecular response

RT‐qPCR assessments were carried out at the Reference Laboratory of each participating Center, according to ELN Guidelines. At each time point scheduled for the MRD monitoring, 10 ml of peripheral blood was sampled for RT‐qPCR analysis. Molecular response (MR) by RT‐qPCR was defined according to the latest laboratory recommendations and using ABL1 as reference gene. Measurable MR was assigned following the international scale (IS) and scored MR^3.0^ if ≤0.1% BCR‐ABL1%IS, MR^4.0^ if ≤0.01% BCR‐ABL1%IS, MR^4.5^ if ≤0.0032% BCR‐ABL1%IS, and MR^5.0^ if ≤0.001 BCR‐ABL1%IS. Minimum sum of ABL1 reference gene transcripts, irrespective of whether BCR‐ABL1 was detected or not, 10.000, 32.000, and 100.000 for MR^4.0^, MR^4.5^, and MR^5.0^, respectively. The participating Reference Labs belonged to the Gruppo Italiano Malattie Ematologiche dell’Adulto (GIMEMA) Labnet and accredited by the GIMEMA Labnet Quality Committee to release the results of RT‐qPCR analysis, since certified for the quantification of BCR‐ABL1 according to the IS, as recommended by the International Experts Panel's Guide Lines.^26^

### 2.3 Procedures for HRQoL assessment

Health‐related quality of life (HRQoL) was assessed with the EORTC Quality of Life Questionnaire‐Core 30 (EORTC QLQ‐C30)^27^ and its QLQ‐CML24 ^28^ and the QLQ‐ELD‐14 ^29^ modules. During the first year (i.e., when treatment schedule between arms was not different, 1 month ON/OFF), the protocol stipulated that HRQoL had to be assessed at baseline and then at 3, 6, and at 12 months. Afterwards, HRQoL was assessed at 18, 24, 30, and 36 months and these time points were chosen to maximize the sensitivity to possible effects of treatments being tested.

### 2.4 Statistical analysis

The HRQoL compliance for each time point was calculated as the percentage of returned questionnaires out of those expected from patients still on study. Proportions, means, standard deviation, and medians were used to summarize patients’ characteristics. We used uni‐ and multivariable linear regression analysis to estimate the association of baseline global health status/QoL with key sociodemographic and clinical factors such as age, gender, comorbidities (at least two vs. one or less), type of TKI (first vs. second generation), and the duration of therapy with TKIs at study entry (months). We also used a linear mixed model for repeated measures to estimate mean HRQoL trajectories over time, testing the null hypothesis of no change from baseline by an overall F‐test. We used the same approach to estimate the mean HRQoL trajectories by sex, testing the null hypothesis of no difference between men and women. For descriptive purposes, we also assessed the prevalence of clinically important problems and symptoms by gender, as defined as in previously published work.^30^ All statistical tests were two‐sided, with statistical significance set as α = 0.05. Due to the exploratory nature of the analyses, we did not adjust for multiple testing.

## 3 RESULTS

### 3.1 Interim report

In this first interim report, the patients who, by intention to treat, have completed the first year of the study were evaluated. Since the treatment intermittent schedule is not different during the 1^st^ year of therapy for both the patients randomized in the “fixed” and in the “progressive” arms, it is expected that no differences will be found up to the end of the 1^st^ year in the two arms, but information on loosing of MR^3.0^/MR^4.0^ molecular response and HRQoL can be obtained.

### 3.2 Treatment and molecular results

Up to December 2018, 185 patients have been enrolled by 26 Italian Hematological Centers (first patient randomized in July 2015) and 166/185 patients (90%) completed the first year of follow up in the OPTkIMA study. Table 1 reports the most important clinical and biological characteristics of the 185 patients. The median age was 71 years (range 60–89) and 61% of the patients belonged to the Sokal intermediate‐/high‐risk group. A total of 140/185 (76%), 25/185 (13%), and 20/185 (11%) patients were receiving IM, NIL, and DAS, at the time of enrollment, respectively. Overall, 99/185 (54%) and 86/185 (46%) patients have been randomized in the “fixed” and “progressive” arms, respectively.

**TABLE 1:** Clinical and biological characteristics of the 185 patients enrolled in OPTkIMA trial

Variable	Total (n = 185)	Fixed (n = 99 – 54%)	Progressive (n = 86 – 46%)	*p*
M/F	106/79 (57% / 43%)	51/48 (52% ‐ 48%)	55/31 (64% ‐ 36%)	0.10
Median age (range)	71 (60–89)	70 (60–89)	72.5 (60–85)	**0.02**
Type of transcript	185	99	85	0.24
b3a2	121 (65%)	61 (62%)	59 (69%)	
b2a2	64 (35%)	38 (38%)	26 (31%)	
Sokal				0.09
L	73 (40%)	37 (37%)	36 (42)	0.09
I	82 (44%)	42 (42%)	41 (48%)	
H	30 (16%)	21 (21%)	9 (10%)	
TKI				0.66
IMA median dose (range)	140 (76%) 400 mg (100–600)	72 (73%) 400 mg (100–600)	68 (79%) 400 mg (100–400)	0.54
NILO median dose (range)	25 (13%) 600 mg (200–800)	15 (15%) 600 mg (200–800)	10 (12%) 600 mg (200–600)	0.32
DAS median dose (range)	20 (11%) 100 mg (50–100)	12 (12%) 100 mg (50–100)	8 (9%) 100 mg (50–100)	0.12
Median duration of TKI (mo)	87.5 (24–245)	85 (24–245)	90 (24–194)	0.79
Molecular response at enrollment				0.48
MR^3.0^	37 (20%)	18 (18%)	19 (22%)	
MR^4.0^	146 (79%)	78 (79%)	67 (78%)	
> MR^4.0^	2 (1%)	2 (2%)	0 (0%)	
Pts with at least 2 comorbidities	121 (65%)	58 (58%)	63 (73%)	0.07
Pts with at least 2 drugs other than TKI	117 (63%)	60 (61%)	57 (66%)	0.10

L = Sokal Low risk; I = Sokal Intermediate risk; H = Sokal High risk; IMA = imatinib; NILO = nilotinib; DAS = dasatinib.

A total of 47/166 patients (28%) abandoned the intermittent treatment during the 1^st^ year. The reasons of intermittent schedule discontinuation were as follows: informed consent withdrawn (4/166–2%), second cancer (4/166–2%), and loss of MR^3.0^ (39/166–23.5%) (Table 2). No patient progressed to AP/BP.

**TABLE 2:** Patients and causes of OPTkIMA discontinuation in the 1^st^ year of intermittent treatment 1 month ON and 1 month OFF

	TOT	IC withdrawn	Second Cancer	Loss of MR3.0
3°Month	14	3	1	10
6°Month	21	1	1	19
9°Month	4	0	1	3
12°Month	8	0	1	7
TOT	47/166 (28%)	4/166 (2%)	4/166 (2%)	39^a^/166 (23%)

^a^ At the time of enrollment: 17 in MR^3.0^ and 22 in MR^4.0^.

**FIGURE 1:**
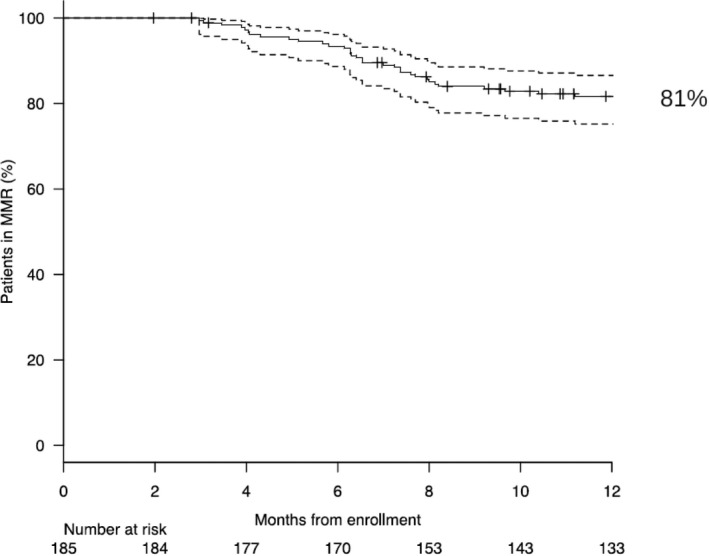
Probability of maintaining MR3.0 at 1st year of OPTkIMA

Considering these last 39 patients who lost MR^3.0^ in the 1^st^ year, 22 and 17 were in MR^4.0^ and MR^3.0^ when they were enrolled into the study, respectively. Thus, the probability of maintaining the MR^3.0^ while on OPTkIMA was 81% at 1 year (95% CI 0.75–0.87) (Figure 1). Moreover, of the 166 patients who completed the first year of OPTkIMA, 136 (82%) and 30 (18%) were in MR^4.0^ and MR^3.0^ at baseline, respectively. As a consequence, 22/136 (16%) and 17/30 (57%) patients in MR^4.0^ and MR^3.0^ at baseline lost the molecular response during the 1st year (*p* < 0.00001). The impact of the most important clinical and biological variables (age, gender, disease risk according to Sokal, EURO, and EUTOS score, time from diagnosis to enrollment, type of BCR‐ABL transcript, dose of TKI, duration of TKI, depth of molecular response, and duration of MR^3.0^) on the probability to maintain the MR^3.0^ was analyzed by univariate analysis. The only factor associated with a higher probability to maintain the MR^3.0^ was a duration of MR^3.0^ greater than 3 years [HR 0.23, 95%CI 0.10–0.61), *p* = 0.0025] (Table 3). All the 39 patients resumed the same TKI continuously and all but 2 (95%) obtained at least the MR^3.0^ response, within 6 months. The mutational analysis was performed by denaturing high‐performance liquid cromatography (DHPLC) in all the cases and in 2/39 patients (5%) an ABL mutation was detected (D363Y and Y320C). Both of these 2 patients did not re‐achieve the MR^3.0^ with imatinib: one shifted to nilotinib after 3 months and is currently in follow up; the other one died for progressive rheumatologic disease not in MR^3.0^.

**TABLE 3:**
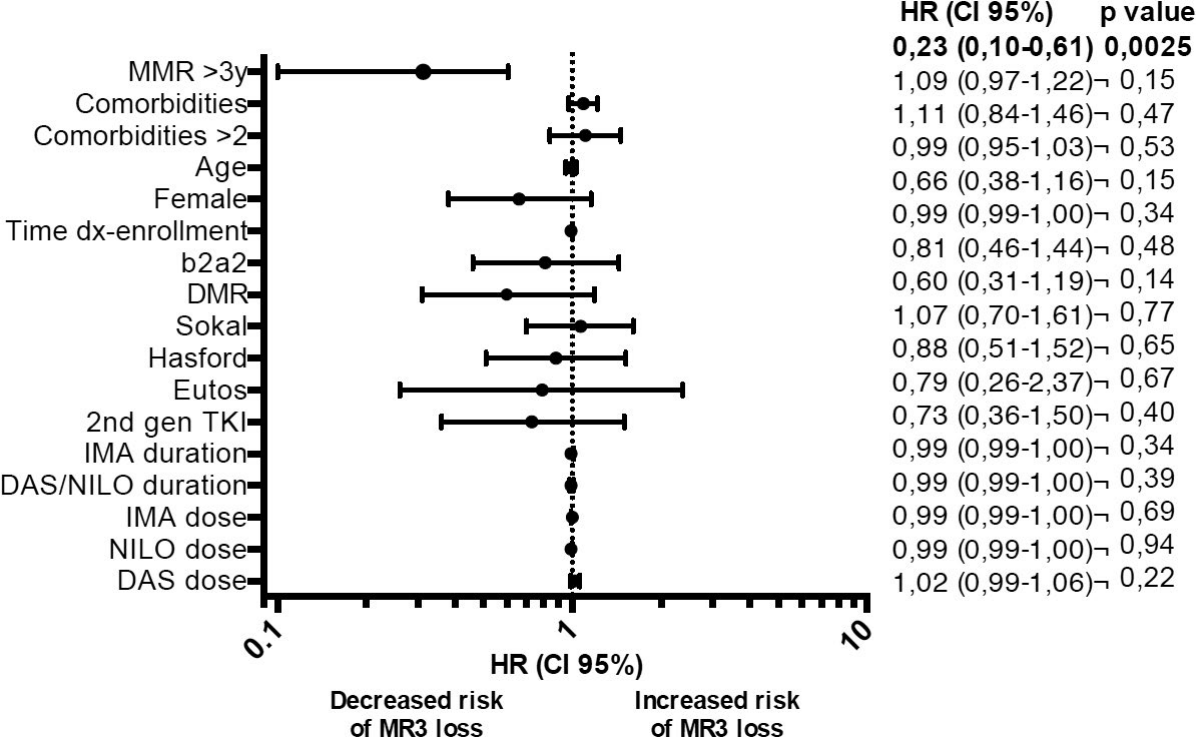
Forest plot analysis on the impact of the clinical and biological variables on the probability of MR^3.0^ loss—Univariate analysis

### 3.3 Treatment and tolerance

The adverse events registered in the electronic CRFs and managed according to the published ELN guidelines^31^ are reported in Table 4. Overall, the intermittent treatment was well tolerated, with 6 serious adverse events (2 appendicitis, 1 peri‐anal abscess, 1 heart‐failure, 1 hip fracture, and 1 severe artheriopathy) and 27 nonsevere adverse, of which 13 were reported in the first 9 months of the study time and 14 at the 12^th^ month. None of these events were treatment related. According to the data reported in the electronic CRFs, none of the patients experienced the TKI withdrawn syndrome.

**TABLE 4:** AEs reported during the 1^st^ year of OPTkIMA Study

AE	Severe	Month of OPTkIMA	ON/OFF
Artheriopathy	Yes	1st	ON
Heart failure	Yes	2nd	OFF
Appendicitis	Yes	4th	OFF
Appendicitis	Yes	11th	ON
Hip fracture	Yes	11th	ON
Peri‐anal abscess	Yes	12th	OFF
Ascites	No	1st	ON
Arthritis	No	2nd	OFF
Chills	No	2nd	OFF
Cramps	No	3rd	ON
Hand pain	No	3rd	ON
Chills	No	5th	ON
Legs edema	No	6th	OFF
Atrial Fibrillation	No	6th	OFF
Inguinal Hernia	No	7th	ON
Conjunctiva bleeding	No	9th	ON
Hypotension	No	9th	ON
Dyspnea	No	9th	ON
Arthritis	No	9th	ON
Pneumonia	No	10th	OFF
Peri‐orbital edema	No	10th	OFF
Bronchitis	No	10th	OFF
Diarrhea	No	11th	ON
Influenza	No	12th	OFF
Fever	No	12th	OFF
Itch	No	12th	OFF
Fever	No	12th	OFF
Drowsiness	No	12th	OFF
Fever	No	12th	OFF
Post‐vitreous detachment	No	12th	OFF
Diarrohes	No	12th	OFF
Cramps	No	12th	OFF
Hypertension	No	12th	OFF

### 3.4 Update beyond the 1^st^ year

After the 1^st^ year of intermittent treatment (1 month ON/OFF), 119 patients entered the 2^nd^ year, of whom 63 (54%) and 56 (47%) belonged to the “fixed “and “progressive” arm, respectively. Overall, 105/119 patients (88%) completed the 2^nd^ year, of whom 59 (56%) in the “fixed” and 46 (44%) in the “progressive” arm.

Of 63 patients randomized to the “fixed” arm who entered the 2^nd^ year, 4 (6%) discontinued the intermittent schedule because of death for second cancer (2 cases) and senectus (2 cases). No patient lost the MR^3.0^.

### 3.5 Health‐related quality of Life (HRQoL) results

#### 3.5.1 Compliance and baseline QoL Profile

**FIGURE 2:**
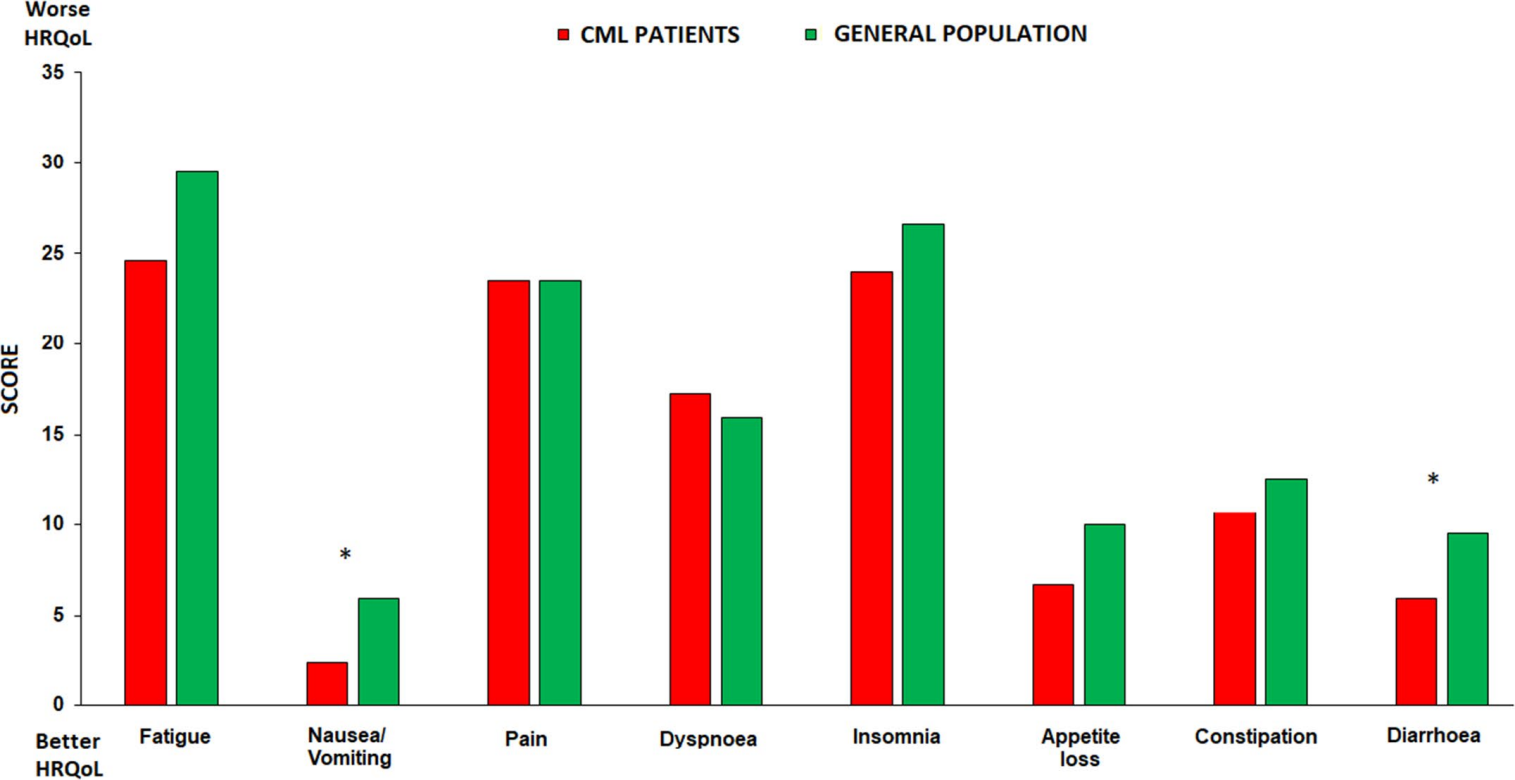
Baseline symptom profile of OPTkIMA patients versus sex and age matched peers from general population. *Legend*: * = clinically meaningful difference. The figure represents the age‐sex adjusted mean level of symptom burden per group

**FIGURE 3:**
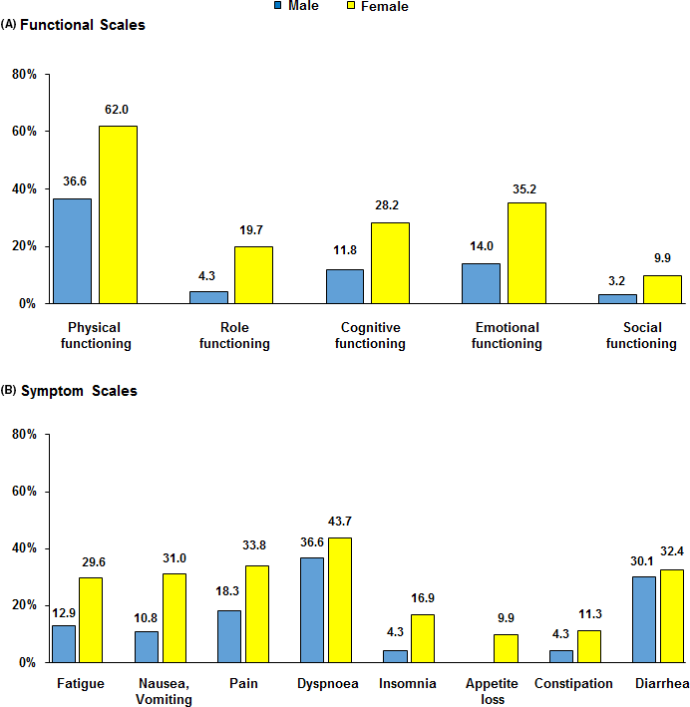
Prevalence of clinically important problems and symptoms by gender. (A) Functional scales, (B) Symptom scales. Legend: Clinically important problems and symptoms (Giesinger et al. J Clin Epidemiol. 2019 Oct 19;118:1–8)

Compliance with HRQoL questionnaires was as follows: 96%, 91%, 92%, and 80% at baseline, 3, 6, and 12 months, respectively. As reported in Figure 2, baseline symptoms profile of OPTkIMA patients generally showed better reported symptoms with respect to gender‐ and age‐matched peers from the general population.^32^, ^33^ Female gender was the only factor independently associated with worse baseline HRQoL in multivariable analysis (*p* <.0001), while controlling for other key potential confounding factors, including age, number of comorbidities, type of TKI, and duration of previous treatment (Table 5). The prevalence of clinically relevant problems was higher in female patients across all EORTC QLQ‐C30 scales with the largest difference found for physical functioning, being 36.6% and 62% for male and female patients, respectively. Details are reported in Figure 3.^30^

**TABLE 5:** Multivariable regression analysis of baseline Global Quality of Life (EORTC QLQ‐C30)

Variables	Univariate analysis		Multivariate analysis	
	Estimate (95% CI)	*p*‐value	Estimate (95% CI)	*p*‐value
Age (y)	−0.28 (−0.70; 0.14)	0.192	−0.23 (−0.63; 0.18)	0.273
Being female	−11.96 (−17.82; −6.11)	**<0.0001**	−12.51 (−18.32; −6.70)	**<0.0001**
At least 2 comorbidities	−5.16 (−11.24; 0.92)	0.096	−5.28 (−11.17; 0.61)	0.079
2^nd^‐generation TKI^a^	−2.20 (−9.40; 5.01)	0.548	−0.31 (−7.33; 6.7)	0.930
TKI duration (months)	0.07 (0.01; 0.13)	**0.036**	0.06 (0.00; 0.13)	0.062

The bold values represent the significant parameters associated with global HRQoL.

^a^ Nilotinib or dasatinib (reference category is the first‐generation TKI imatinib). CI, confidence interval.

#### 3.5.2 Longitudinal QoL profile

**FIGURE 4:**
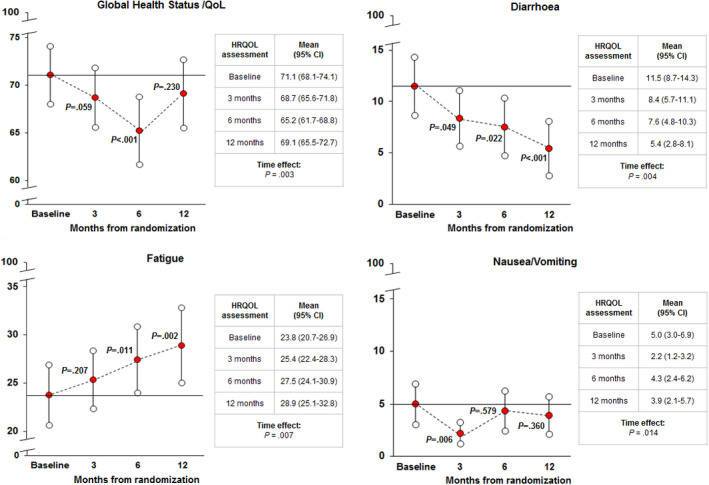
Trajectories of change in quality of life outcomes for selected EORTC QLQ‐C30 scales. Legend: For the global QoL scale higher scores indicate better QoL outcomes, whereas lower scores indicate worse QoL outcomes. For diarrhoea, fatigue, and nausea/vomiting higher scores indicate higher symptom severity, whereas lower scores indicate lower symptom severity. P‐values refer to change from baseline

**FIGURE 5:**
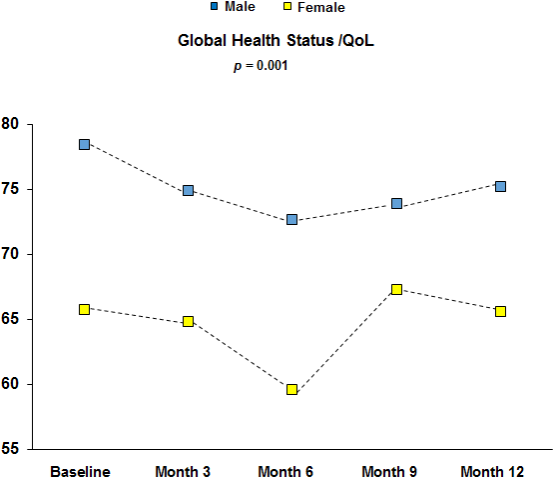
Trajectories of gender differences in Global Quality of Life up to 12 months

Statistically significant improvements over time were found for diarrhea at 6 (*p* = 0.022) and 12 months (*p* = 0.022), with mean score decreasing from 11.5 points at baseline to 5.4 points at 12 months. These changes were also clinically meaningful across all time points (Figure 4). Nausea and vomiting scale also improved at 3 months (*p* =0.006) and then returned to baseline levels (Figure 4). There was a statically significant worsening in fatigue severity at 6 (*p* = 0.001) and 12 months (*p* = 0.022) (Figure 4). Global HRQoL also decreased at 6 months (*p* < 0.001) but then returned to baseline levels at 12 months (Figure 4). However, global HRQoL over time was statically significantly different (*p* = 0.001) by gender with female patients reporting worse outcomes during the 12 months of observation (Figure 5). No other scales of the EORTC QLQ‐C30 showed a statistically significant difference from baseline at any time point.

## 4 DISCUSSION

OPTkIMA Study is a phase III Italian randomized trial, started in July 2015, where elderly (≥60 years) CML patients in stable molecular response (MR^3.0^ or MR^4.0^) after ≥2 years of standard treatment with IM, NIL, or DAS are randomized 1:1 to receive “fixed” or “progressive” intermittent TKI administration until MR^3.0^ is lost.

The main objectives of the study are to assess the changing of HRQoL in the patients moving from a continuous to a progressive de‐escalated intermittent TKIs treatment and to find the TKI minimum effective dose able to maintain the major molecular response (MR^3.0^).

It is known that persistent or recurrent low‐grade adverse events (AEs) are common in CML patients during chronic treatment with TKIs. These events alter the patient's HRQoL and adherence to therapy and may cause lifelong comorbidities, especially in the elderly.

This first interim analysis was made to acquire information on HRQoL and on the maintenance of MR^3.0^/MR^4.0^ in those patients of the both arms who received IM, NIL, or DAS “one month ON/OFF” for the 1^st^ year.

Although patients’ accrual was lower than expected, the compliance in reporting the HRQoL questionnaires was extremely satisfying (more than 90% in the first 6 months and 80% at the 12^th^ month). Analyzing the HRQoL, the baseline profile of OPTkIMA patients was better than that of gender‐ and age‐ matched healthy participants (Figure 2).^32^, ^33^ We were surprised of that, and even more by the fact that female was the only factor independently associated with worse baseline HRQoL in multivariable analysis (*p* < 0.0001) (Table 2).

Concerning these findings, we can speculate that elderly patients with CML who are in stable molecular response have a good HRQoL, being confident with their disease, and considering themselves as cancer Survivors could tend to minimize some symptoms associated with aging, that, on the contrary, patients without cancer may consider as important and detrimental for HRQoL. In the second case, the prevalence of clinically relevant symptoms was higher in women (62%) than in men (36.6%), and the largest difference was found in the physical functioning (Figure 3).

Other hematologic and oncologic studies investigating the HRQoL report a worst HRQoL in the female with respect to the male gender,^34^ and different factors have been discussed as possible causes. The modern society strongly leans on elderly women more than elderly men (e.g., daily management of the family, housekeeping, grandchildren management, etc.). As a consequence, a cancer diagnosis, its treatment, as well as the participation in a clinical trial, such as OPTkIMA, may impair the physical and mental functioning of an elderly female patient more significantly than those of elderly male patient.

Monitoring the symptoms, a statistically significant improvement over time was found for diarrhea at 6 (*p* = 0.022) and 12 months (*p* = 0.022) (Figure 4). For other symptoms, such as nausea and vomiting, the scale also improved at 3 months (*p* = 0.006) and then returned to baseline levels (Figure 4). On the contrary, there was a statically significant worsening in fatigue severity at 6 (*p* = 0.001) and 12 months (*p* = 0.022) (Figure 4).

Global HRQoL also decreased at 6 months (*p* < 0.001) but then returned to baseline levels at 12 months (Figure 4).). Once again, when the global HRQoL over time was evaluated, it was statistically significantly different (*p* = 0.001) by gender with female patients reporting worse outcomes during the 12 months of observation (Figure 5).

The interpretation of these data is indeed difficult and intriguing. The amelioration of symptoms such as diarrhea or nausea can be related to the intermittent TKI administration that, particularly in the first months after its initiation, can significantly reduce symptoms associated with the continuous long‐lasting TKI administration (notably the median duration of continuous TKI administration ranges between 85 and 90 months in our patients).

The worsening in the fatigue scale at 6th and 12th month and the decrease in the global HRQoL at 6th month could be induced by the stress in patients who are normally well confident with their disease, its control, and its remission. Furthermore, even though no physician reported in the electronic CRF the typical TKI withdrawn syndrome, it is possible that some mild symptoms associated with the month OFF may have affected the patient's reported outcome. However, we did not observe any significant reduction in hemoglobin level and none of the patients retired consent, suggesting that this lack of “improvement” in HRQol was not significantly deemed by patients.

From the hematologic point of view, our analysis confirmed that the intermittent therapy is effective and safe. During the 1^st^ year, 39/166 patients (23.5%) lost MR^3.0^ and all of them re‐gained the major molecular response within 6 months from resumption of continuous treatment. The probability of MR^3.0^ maintenance while on OPTkIMA at 1 year was 81% (95% CI 0.75–0.87; Figure 1), quite comparable with the 80% MR^3.0^ maintenance at 1 year observed in the previous INTERIM trial.^20^, ^21^

Of note, the depth of MR at baseline (MR^3.0^ and MR^4.0^) had an impact on the probability to maintain the MR while on intermittent schedule. Focusing on the 166 patients who completed the 1^st^ year, 30 and 136 were in MR^3.0^ and MR^4.0^ at baseline; 17/30 (57%) and 22/136 (16%) lost the molecular response during the 1st year, respectively (*p* < 0.00001). The only factor associated with a higher probability to maintain the MR^3.0^ by univariate analysis was a duration of MR^3.0^ greater than 3 years [HR 0.23, 95%CI 0.10–0.61), *p* = 0.0025] (Table 3). The study is ongoing, and after the 1^st^ year of intermittent treatment (1 month ON/OFF), 119 patients entered the 2^nd^ year, 63 in the “fixed”, and 56 in the “progressive” arm, respectively, and none of them lost the MR^3.0^. Only another study explored a policy of TKI dose reduction. It is the DESTINY trial, in which a 50% daily dose reduction of IM in the first year followed by discontinuation of TKI was adopted [DESTINY trial ^35^]. Although DESTINY and OPTkIMA trials seem similar, they have substantial differences. OPTkIMA trial includes elderly patients and is not aimed to treatment permanent discontinuation, but is planned to identify the minimum effective dose of TKI (IM, NIL, and DAS) able to maintain the MR^3.0^, which is a surrogate marker of survival. DESTINY trial enrolled younger patients and a 50% dose de‐escalation for 12 months is scheduled as a bridge to IM discontinuation. This “clinical” selection may be improved by monitoring strictly the slope of the MR and by selecting for TD those patients with a MR slope showing a stability of BCR‐ABL1 transcript levels.^36^

In conclusion, this first interim analysis showed that the intermittent therapy, 1 month ON/OFF, is equally effective and safe across all the three TKIs. It also allowed to acquire basal and relevant information on HRQoL in elderly CML patients moving from standard daily therapy to experimental intermittent treatment. Significant differences in the HRQoL were observed in comparison with healthy peers matched for gender and age and between men and women. The study is ongoing and relevant information on the comparison of HRQoL between the “fixed” and the “progressive” randomization arms will be available in the next future.

## CONFLICTS OF INTEREST

AI—speaker honoraria from Novartis, Pfizer, and Incyte; EA—consultancy and advisory for Novartis, Bristol Myers Squibb, Incyte, and Pfizer; GR—speaker honoraria from Novartis, Pfizer, and Incyte; MB—consultant and receiving honoraria from Novartis, Incyte, and Takeda; DR—speaker honoraria: MSD, Novartis, Gilead; advisory committees: MSD, Janssen, Gilead. All the other authors declare no conflicts of interest.

## AUTHOR CONTRIBUTIONS

DR is the PI of the study. DR, MM, and MB designed the study; DR, MM, AI, EA, MB, SB, and NP analyzed the data and wrote the study; ADV generated the electronic database; all the Authors collected the data; and all the authors revised the final version of the paper and approved it before its submission.

## Data Availability

The data that support the findings of this study are available from the corresponding author upon reasonable request, considering that the study is ongoing.
