# Corrigendum

**DOI:** 10.1002/cam4.3922

**Published:** 2021-05-02

In the article by Malagola et al.,^1^ entitled “Molecular response, quality of life in chronic myeloid leukemia patients treated with intermittent TKIs: First interim analysis of OPTkIMA study,” the authors would like to add a missing author, correct the affiliation of an existing author

Monica Bocchia has been added and should appear as the 24^th^ author. Dr. Bocchia’s affiliation is “Hematology Unit, Department of Medicine, Surgery and Neuroscience, Azienda Ospedaliera Universitaria Senese, University of Siena, Siena, Italy.”

The affiliation for Lara Aprile should be “SC Ematologia, Ospedale S.G.Moscati, Taranto, Italy.”
